# A deep learning algorithm using CT images to screen for Corona virus disease (COVID-19)

**DOI:** 10.1007/s00330-021-07715-1

**Published:** 2021-02-24

**Authors:** Shuai Wang, Bo Kang, Jinlu Ma, Xianjun Zeng, Mingming Xiao, Jia Guo, Mengjiao Cai, Jingyi Yang, Yaodong Li, Xiangfei Meng, Bo Xu

**Affiliations:** 1 Department of Biochemistry and Molecular Biology, National Clinical Research Center for Cancer, Key Laboratory of Cancer Prevention and Therapy, Key Laboratory of Breast Cancer Prevention and Therapy, Ministry of Education, Tianjin Clinical Research Center for Cancer, Tianjin Medical University Cancer Institute and Hospital, Tianjin, 300060 China; 2 Department of Hepatobiliary Oncology, Tianjin Medical University Cancer Institute and Hospital, National Clinical Research Center for Cancer, Tianjin, 300060 China; 3 College of Intelligence and Computing, Tianjin University, Tianjin, 300350 China; 4 Present Address: National Supercomputer Center in Tianjin, Tianjin, 300457 China; 5 Department of Radiation Oncology, First Affiliated Hospital, Xi’an Jiaotong University, Xi’an, China; 6 Department of Radiology, Nanchang University First Hospital, Nanchang, China; 7 Department of Radiology, No.8 Hospital, Xi’an Medical College, Xi’an, China; 8 Present Address: Center for Intelligent Oncology, Chongqing University Cancer Hospital, Chongqing University School of Medicine, Chongqing, China

**Keywords:** COVID-19, Tomography, X-ray computed, Artificial intelligence, Deep learning, Diagnosis

## Abstract

**Objective:**

The outbreak of Severe Acute Respiratory Syndrome Coronavirus 2 (SARS-COV-2) has caused more than 26 million cases of Corona virus disease (COVID-19) in the world so far. To control the spread of the disease, screening large numbers of suspected cases for appropriate quarantine and treatment are a priority. Pathogenic laboratory testing is typically the gold standard, but it bears the burden of significant false negativity, adding to the urgent need of alternative diagnostic methods to combat the disease. Based on COVID-19 radiographic changes in CT images, this study hypothesized that artificial intelligence methods might be able to extract specific graphical features of COVID-19 and provide a clinical diagnosis ahead of the pathogenic test, thus saving critical time for disease control.

**Methods:**

We collected 1065 CT images of pathogen-confirmed COVID-19 cases along with those previously diagnosed with typical viral pneumonia. We modified the inception transfer-learning model to establish the algorithm, followed by internal and external validation.

**Results:**

The internal validation achieved a total accuracy of 89.5% with a specificity of 0.88 and sensitivity of 0.87. The external testing dataset showed a total accuracy of 79.3% with a specificity of 0.83 and sensitivity of 0.67. In addition, in 54 COVID-19 images, the first two nucleic acid test results were negative, and 46 were predicted as COVID-19 positive by the algorithm, with an accuracy of 85.2%.

**Conclusion:**

These results demonstrate the proof-of-principle for using artificial intelligence to extract radiological features for timely and accurate COVID-19 diagnosis.

**Key Points:**

*• The study evaluated the diagnostic performance of a deep learning algorithm using CT images to screen for COVID-19 during the influenza season.*

*• As a screening method, our model achieved a relatively high sensitivity on internal and external CT image datasets.*

*• The model was used to distinguish between COVID-19 and other typical viral pneumonia, both of which have quite similar radiologic characteristics.*

## Introduction

The outbreak of atypical and person-to-person transmissible pneumonia caused by the severe acute respiratory syndrome corona virus 2 (SARS-COV-2, also known as 2019-nCov) has caused a global pandemic. There have been more than 6.1 million confirmed cases of the Corona virus disease (COVID-19) in the world, as of the 1^st^ of June 2020. About 16–21% of people with the virus in China have become severely ill with a 2–3% mortality rate. With the most recent estimated viral reproduction number (R0), in a completely non-immune population, the average number of other people that an infected individual will transmit the virus to stands at about 3.77 [1, 2], indicating that a rapid spread of the disease is imminent. It is crucial to identify infected individuals as early as possible for quarantine and treatment procedures.

The diagnosis of COVID-19 relies on the following criteria: clinical symptoms, epidemiological history and positive CT images, and positive pathogenic testing. The clinical characteristics of COVID-19 include respiratory symptoms, fever, cough, dyspnea, and pneumonia [3–6]. However, these symptoms are nonspecific, as there are isolated cases wherein, for example, in an asymptomatic-infected family, a chest CT scan revealed pneumonia and the pathogenic test for the virus reported a positive result. Once someone is identified as a person under investigation (PUI), lower respiratory specimens, such as bronchoalveolar lavage, tracheal aspirate, or sputum, will be collected for pathogenic testing. This laboratory technology is based on real-time RT-PCR and sequencing of nucleic acids from the virus [7, 8]. Since the outbreak of COVID-19, the efficiency of nucleic acid testing has been dependent on several rate-limiting factors, including the availability and quantity of the testing kits in the affected areas. Moreover, the quality, stability, and reproducibility of the detection kits are questionable. The impact of methodology, disease stages, specimen collection methods, nucleic acid extraction methods, and the amplification system are all determinant factors for the accuracy of the test results. Conservative estimates of the detection rate of nucleic acid are low (30**–**50%) [9], and the tests must be repeated several times in many cases before the results are confirmed.

Another major diagnostic tool for COVID-19 is radiological imaging. The majority of COVID-19 cases have similar features on CT images including ground-glass opacities in the early stage and pulmonary consolidation in the later stage. Occasionally, a rounded morphology and a peripheral lung distribution can also be observed [4, 10]. Although typical CT images may help early screening of suspected cases, the images of various viral pneumonia are similar, and they overlap with other infectious and inflammatory lung diseases. Therefore, it is difficult for radiologists to distinguish COVID-19 from other viral pneumonia.

AI involving medical imaging-based deep learning systems has been developed in image feature extraction, including shape and spatial relation features. Specifically, the convolutional neural network (CNN) has shown promising results in feature extraction and learning. CNN has been used to enhance low-light images from high-speed video endoscopy, with limited training data from just 55 videos [11]. In addition, CNN has been applied to identify the nature of pulmonary nodules via CT images, the diagnosis of pediatric pneumonia via chest X-ray images, for precise automation and labelling of polyps during colonoscopy videos, and cystoscopy image recognition extraction from videos [12–15].

There are several features for identifying viral pathogens on the basis of imaging patterns, which are associated with their specific pathogenesis [16]. The hallmarks of COVID-19 patterns are bilateral distributions of patchy shadows and ground-glass opacity in the early stages. As the disease progresses, multiple ground glass and infiltrates appear in both lungs [6]. These features are quite similar to typical viral pneumonia with only slight differences, which are difficult for radiologists to distinguish. Based on this, we believe that CNN might help us identify unique features that might be difficult using visual recognition.

Hence, our study evaluates the diagnostic performance of a deep learning algorithm using CT images to screen for COVID-19 during the influenza season. To test this hypothesis, we retrospectively enrolled 1065 CT images of pathogen-confirmed COVID-19 cases along with previously diagnosed typical viral pneumonia. Our results demonstrate the proof-of-principle using the deep learning method to extract radiological graphical features for COVID-19 diagnosis.

## Methods and materials

### Retrospective collection of datasets

We retrospectively collected CT images from 259 patients, in which the cohort included 180 cases of typical viral pneumonia and the other 79 cases from three hospitals with confirmed nucleic acid testing of SARS-COV-2. In addition, we enrolled 15 additional COVID cases, in which the first two nucleic acid tests were negative in initial diagnoses. The hospitals providing the images were the Xi’an Jiaotong University First Affiliated Hospital (center 1), Nanchang University First Hospital (center 2), and Xi’an No.8 Hospital of Xi’an Medical College (center 3). All CT images were reconfirmed before sending for analysis. This study is in compliance with the institutional review board of each participating institute. Informed consent was exempted by the IRB because of the retrospective nature of this study.

### Delineation of ROIs

To establish a binary model for distinguishing COVID-19 and typical pneumonia, we drew the region of interest (ROI) as the input images for the training and validation cohorts. We sketched the ROI from the CT images based on the features of COVID-19, such as small patchy shadows and interstitial changes in the early stage, multiple ground glass and infiltrates in both lungs in the progression stage, and delineated the ROIs on the CT images of other typical viral pneumonia, such as pseudo cavity, enlarged lymph nodes, and multifocal GGO as the control. We manually delineated the ROI based on the typical features of pneumonia and all the lesion layers were determined to be the input into the model. The ROIs were divided into three cohorts: one training cohort (*n* = 320 from center 1), one internal validation cohort (*n* = 455 from center 1), and one external validation cohort (*n* = 290 from centers 2 and 3). For an ROI, it was sized approximately from 395 × 223 to 636 × 533 pixels.

### Overview of the proposed architecture

**Fig. 1:**
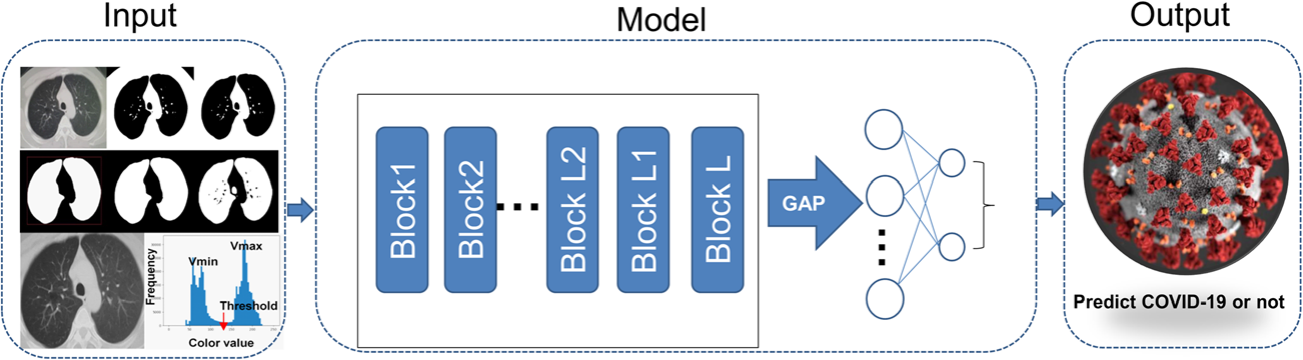
ROI images extraction and deep learning (DL) algorithm framework. ROI images were extracted by the CV model and then trained using a modified inception network to extract features. The full connection layer then performs classification and prediction

Our systematic pipeline for the prediction architecture is depicted in Fig. 1. The architecture consists of three main processes: (1) preprocessing of input images; (2) feature extraction of ROI images and training; and (3) classification with two fully connected layers and prediction of binary classifiers. We performed transfer learning, which involved training with a predefined model using the well-known GoogleNet Inception v3 CNN [16]. The network was already trained on 1.2 million color images from ImageNet that consisted of 1000 categories before learning from the lung radiographs in this study [17]. The entire neural network can be roughly divided into two parts: the first part used a pre-trained inception network to convert image data into one-dimensional feature vectors, and the second part used a fully connected network, mainly for classification prediction. The ROI images from each case were preprocessed and input into the model for training. The number of various types of pictures in the training set is equal, with a total of 320 images. The remaining CT images of each case were used for internal validation. In each iteration of the training process, we fetched a batch of images from the training dataset. The following parameters were used for training: we trained for 15,000 epochs, the initial learning rate of the pre-trained model was 0.01, and it was automatically adjusted with training; furthermore, we used adaptive moment estimation gradient descent for optimization.

### Image preprocessing and feature extraction

Based on the characteristic signs of pneumonia, ROI images were defined as inflammatory lesions and extracted by our computer vision (CV) model as per the following steps:

1. The image was converted to grayscale.
2. Grayscale binarization: As using the OSTU method directly causes the threshold selection failure in the case of multi-peaks, the selection of the binarization threshold in this study was based on the statistics of all pixel frequency histograms of the gray color values Vmin (80) and Vmax (200). The minimum frequency was selected in the selection interval as a threshold, and the interval of frequency statistics was five.
3. Background area filling: The flood filling method was used to expand the image by one black pixel, and the black pixels near the border were filled with white.
4. Reverse color: All the contour areas of the image were determined, and the two largest contour areas were considered as the two lung areas.
5. The smallest bounding rectangle of the lung area was considered the ROI frame, and the original image was cropped to obtain the ROI images.

To obtain more reproducible CT features, the pixel spacing of each CT image acquired from different hospitals and scanners was set to 299 × 299 pixels. The size was not unified. To improve the reliability of the model, the ROI images were processed to a fixed 299 × 299 pixel size, and then the lung contour was precisely delineated to ensure the practicality of the model. The delineated ROIs were obtained for the classification model building. We modified the typical inception network and fine-tuned the modified inception (M-inception) model with pre-trained weights. The size of the input layer of M-inception was 299 × 299 × 3, compatible with the ImageNet. We mapped the gray mode (one channel) to this dimension (299 × 299 × 3, where each channel value of RGB is equal to the gray mode value) to form a virtual RGB format image in the first layer. During the training phase, the original inception part was not trained, and we only trained only the modified part. The architecture of the M-inception is shown in Table 1. The difference between the inception and M-inception model is found in the last of the fully connected layers. We reduced the dimension of the features before it was sent to the final classification layer. The training dataset consisted of all the aforementioned patches. The inception network is shown in Table 1.

**Table 1:** The architecture of M-inception

Inception part	Layer	Patch size/stride or remarks
	Conv	3 × 3/2
	Conv	3 × 3/1
	Conv padded	3 × 3/1
	Pool	3 × 3/2
	Conv	3 × 3/1
	Conv	3 × 3/2
	Conv	3 × 3/1
	Inception	3x, 5x, 2x
	Pool	8 × 8
	Linear	Logits
	Softmax	Classifier
Modified part	Fc1	$$ \left[\begin{array}{c}\mathrm{Batchnorm}\\ {}\mathrm{dropout}(0.5)\\ {}512\mathrm{d}\ \mathrm{Linear}\end{array}\right] $$
	Fc2	$$ \left[\begin{array}{c}\mathrm{Batchnorm}\\ {}\mathrm{dropout}(0.5)\\ {}2\mathrm{d}\ \mathrm{Linear}\end{array}\right] $$

### Prediction

After generating the features, the final step was to classify the pneumonia based on those features. An ensemble of classifiers was used to improve the classification accuracy. In this study, we adopted end-to-end learning to ensure model convergence.

### Performance evaluation metrics

We compared the classification performance using several metrics, such as accuracy, sensitivity, specificity, area under the curve (AUC), positive predictive value (PPV), negative predictive value (NPV), F1 score, and Youden index [18, 19]. TP and TN represent the number of true-positive and true-negative samples, respectively. FP and FN represented the number of false-positive and false-negative samples, respectively. Sensitivity measures the ratio of positives that are correctly discriminated. Specificity measures the ratio of negatives that are correctly discriminated. AUC is an index used to measure the performance of the classifier. NPV was used to evaluate the algorithm for screening, and PPV represents the probability of developing a disease when the diagnostic index is positive. The Youden index is the determining exponent of the optimal bound. The F1 score was a measure of the accuracy of a binary model. Additionally, the performance was evaluated with F-measure (F1) to compare the similarity and diversity of performance. The Kappa value measures the agreement between the CNN model prediction and the clinical report [20].

## Results

### Algorithm development

**Fig. 2:**
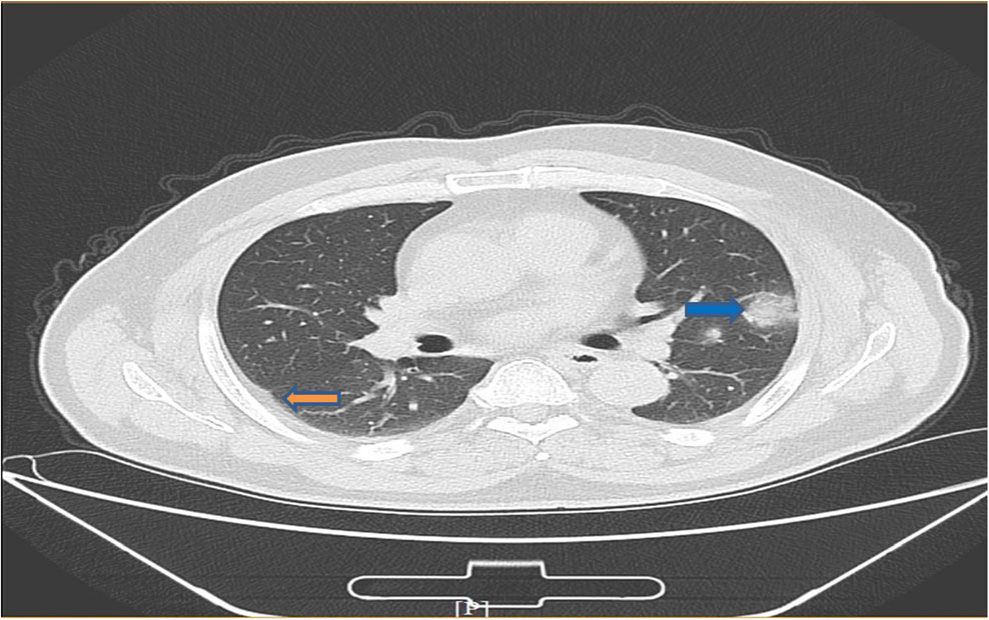
An example of COVID-19 pneumonia features. The blue arrow points to ground-glass opacity, and the orange arrow indicates the pleural indentation sign

**Fig. 3:**
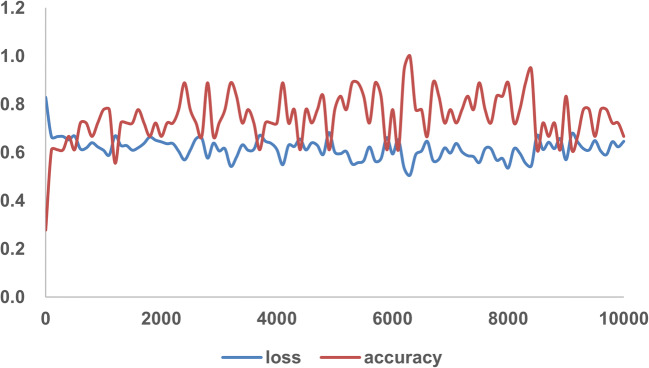
Training loss curves and accuracy of the model. The loss curve and accuracy tend to be stable after descending, indicating that the training process converges

To develop a deep learning algorithm for the identification of viral pneumonia images, we initially enrolled 259 patients, out of which the cohort included 180 cases of typical viral pneumonia, diagnosed before the COVID-19 outbreak. These patients were termed COVID-19 negative in the cohort. The other 79 cases were from the three hospitals with confirmed nucleic acid testing of SARS-COV-2, termed COVID-19 positive. Two radiologists were asked to review the images and sketched 1065 representative images (740 for COVID-19 negative and 325 for COVID-19 positive) for analysis (Fig. 2 is shown as an example). These images were randomly divided into training set and validation set. The model training was iterated for 15,000 epochs with an initial learning rate of 0.01. A total of 320 images (160 images from COVID-19 negative and 160 images from COVID-19 positive) were obtained to construct the model. To test the stability and generalization of the model, 455 images (COVID-19 negative 360 images and COVID-19 positive 95 images) were obtained for internal validation from center 1 and 290 images (COVID-19 negative 220 images and COVID-19 positive 70 images) were obtained from centers 2 and 3 for external validation. The training loss curve and accuracy are shown in Fig. 3. The model was constructed and the validated accuracy was measured for every 100 steps to adjust the super parameter during the training process. Both the accuracy and loss curves tended to be stable.

### Deep learning performance

**Fig. 4:**
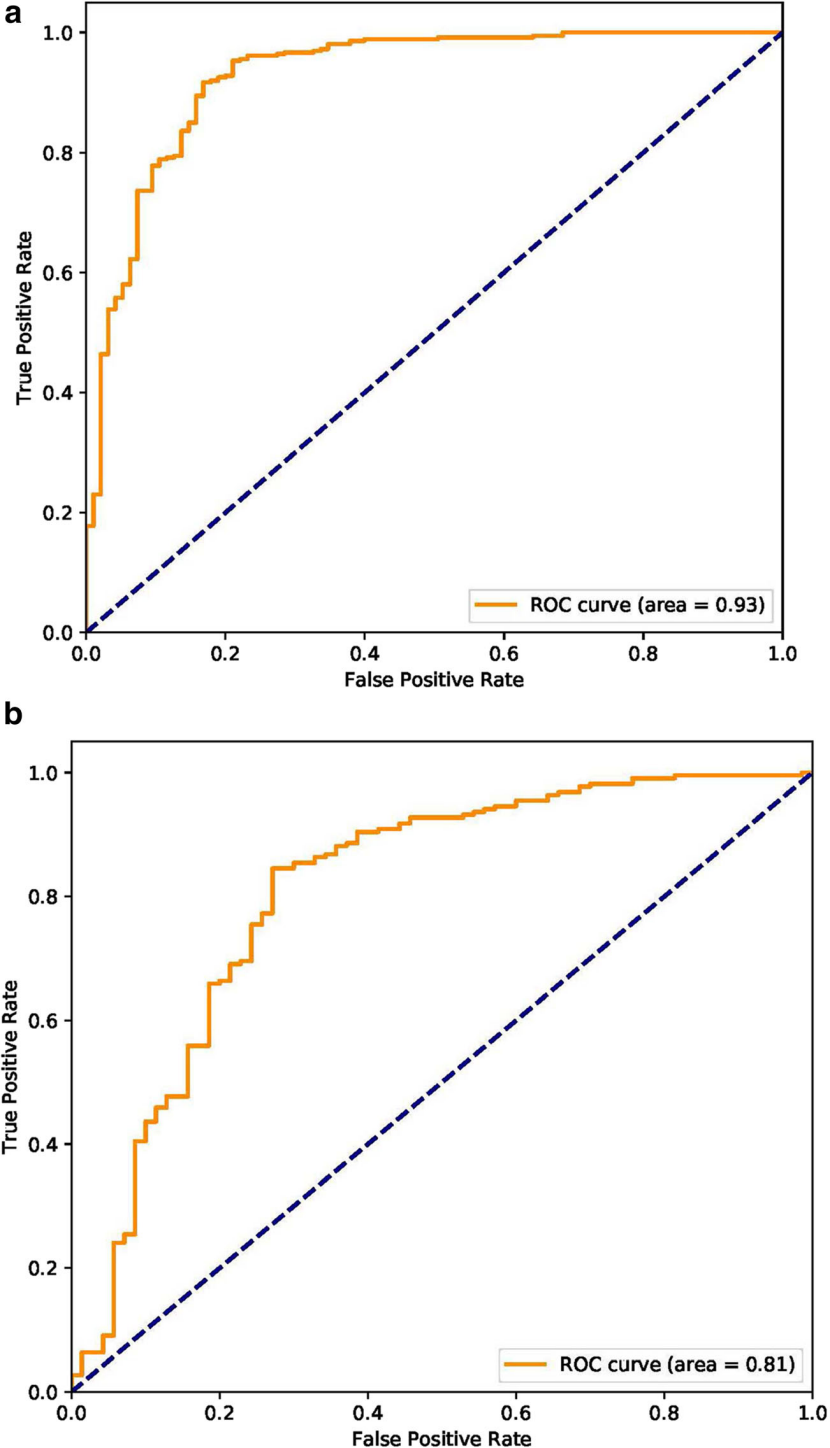
Receiver operating characteristic plots for COVID-19 identification for the deep learning (inception) algorithm. **a** Internal validation. **b** External validation

The algorithm yielded an AUC of 0.93 (95% CI, 0.90 to 0.96) in internal validation and 0.81 (95% CI, 0.71 to 0.84) in the external validation based on the certain CT images (Fig. 4). Using the maximized Youden index threshold probability, the sensitivity was 0.88 and 0.83, specificity was 0.87 and 0.67, accuracy was 89.5% and 79.3%, negative prediction values were 0.95 and 0.90, the Youden indexes were 0.75 and 0.48, and the F1 scores were 0.77 and 0.63 for the internal and external datasets, respectively (Table 2). The kappa values were 0.69 and 0.48 for internal and external validation in certain CT images, respectively, indicating that the prediction of COVID-19 from the CNN model is a highly consistent with the pathogenic testing results. Furthermore, we performed an external validation based on multiple images from each patient. The accuracy was 82.5%, the sensitivity 0.75, the specificity 0.86, the PPV 0.69, the NPV 0.89, and the kappa value was 0.59.

**Table 2:** Deep learning algorithm performance

Performance metric	Internal	External
AUC (95% CI)	0.93 (0.86 to 0.94)	0.81 (0.71 to 0.84)
Accuracy, %	89.5	79.3
Sensitivity	0.88	0.83
Specificity	0.87	0.67
PPV	0.71	0.55
NPV	0.95	0.90
Kappa*	0.69	0.48
Youden index	0.75	0.50
F1 score^ǂ^	0.77	0.63

*Measures the agreement between the CNN model prediction and the clinical diagnosis. ^ǂ^Measures the accuracy of the CNN model

### Comparison of AI with radiologist prediction

At the same time, we asked two skilled radiologists to assess the 745 images for a prediction. Radiologist 1 achieved an accuracy of 55.8% with a sensitivity of 0.71 and specificity of 0.51, and radiologist 2 achieved a similar accuracy of 55.4% with a sensitivity of 0.73 and specificity of 0.50 (Table 3). These results indicate that it was difficult for radiologists to make predictions of COVID-19 with eye recognition, further demonstrating the advantage of the algorithm proposed in this study.

**Table 3:** Performance metrics for the CNN model versus skilled radiologists

Performance metric	Internal	External (based on ROI)	External (based on patients)	R1	R2
Accuracy, %	89.5	79.3	82.5	55.8	55.4
Sensitivity	0.88	0.83	0.75	0.71	0.73
Specificity	0.87	0.67	0.86	0.51	0.5
PPV*	0.71	0.55	0.69	0.29	0.29
NPV^ǂ^	0.95	0.90	0.89	0.86	0.86
F1 score	0.77	0.63	0.72	0.41	0.42
Kappa	0.69	0.48	0.59	0.15	0.15
Youden index	0.75	0.50	0.61	0.22	0.23

*Positive predictive value; ^ǂ^Negative predictive value

### Prediction of COVID-19 on CT images from pathogenic-negative patients

**Fig. 5:**
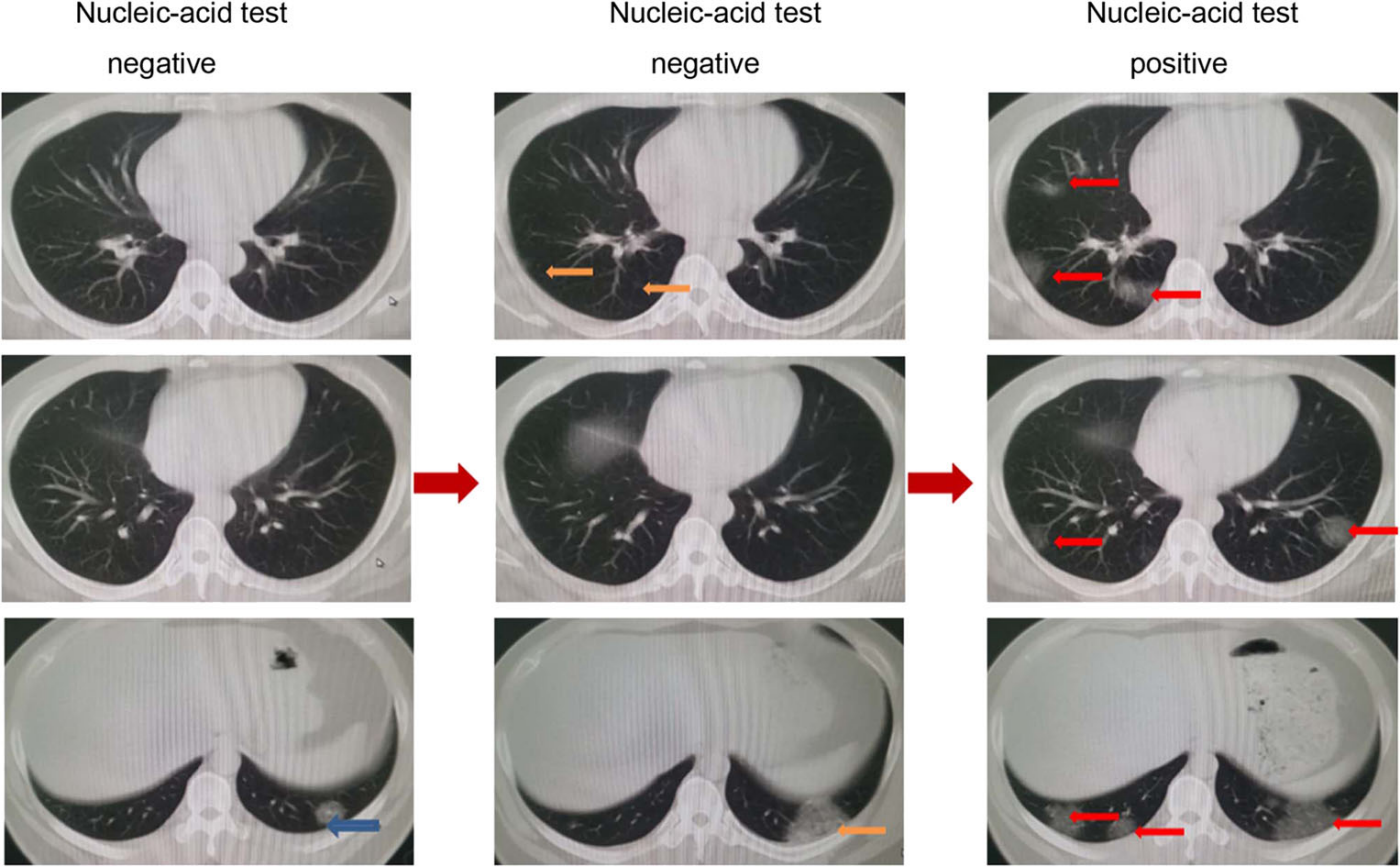
Representative images from a COVID-19 patient with two negatively reported nucleic acid tests at earlier stages and one final positively reported test at a later stage. On the left, only one inflammatory lesion (blue arrow) can be seen near the diaphragm. In the middle, lesions (orange arrows) were found at two levels of images. On the right are the images captured on the ninth day after admission. The inflammation continued to progress, extending to both lungs (red arrows), and the nucleic acid test showed positivity

Because high false-negative results were frequently reported from nucleic acid testing, we aimed to test whether the algorithm could detect COVID-19 when the pathogenic test was negative. To achieve this goal, we enrolled an additional 15 COVID-19 cases, in which the initial two nucleic acid tests were negative, and for the third test, they became positive. These CT results were taken on the same day as the nucleic acid tests (Fig. 5). Interestingly, we found that 46 out of the 54 images, when nucleic acid test results were negative, were predicted as COVID-19 positive by the algorithm, with an accuracy of 85.2%. These results indicate that the algorithm has high value serving as a screening method for COVID-19.

## Discussion

Monitoring and timely identification of PUIs is essential to ensure appropriate triaging of staff for duty, further evaluation, and follow-up. Owing to the limitations of nucleic acid–based laboratory testing, there has been a critical need for faster alternatives that can be used by front-line health care personnel for quickly and accurately diagnosing the disease. In this study, we developed an AI program by analyzing representative CT images using a deep learning method. This is a retrospective, multicenter, diagnostic study using our modified inception migration neuro network, which has achieved an overall accuracy of 89.5%. Moreover, the high performance of the deep learning model we developed in this study was tested using external samples with 79.3% accuracy. More importantly, as a screening method, our model achieved a relatively high sensitivity of 0.88 and 0.83 on internal and external CT image datasets, respectively. Furthermore, the model achieved better performance for certain people, with an accuracy of up to 82.5%. Notably, our model was used to distinguish between COVID-19 and other typical viral pneumonia, both of which have quite similar radiological characteristics. During the current COVID-19 global pandemic, the CNN model can, therefore, potentially serve as a powerful tool for COVID-19 screening.

It is important to note that our model aims to distinguish between COVID-19 and other typical viral pneumonia, both of which have similar radiological characteristics. We compared the performance of our model with that of two skilled radiologists, and our model showed much higher accuracy and sensitivity. These findings demonstrate the proof-of-principle that deep learning can extract CT image features of COVID-19 for diagnostic purposes. Using the supercomputer system, each case took only approximately 10 s, and it can be performed remotely via a shared public platform. Therefore, further development of this system can significantly shorten the diagnosis time for disease control. Our study represents the first study to apply AI technologies to CT images for effective screening of COVID-19.

The gold standard for COVID-19 diagnosis has been nucleic acid–based detection for the existence of specific sequences of the SARS-COV-2 gene. While we still value the importance of nucleic acid detection in the diagnosis of SARS-COV-2 infection, it must be noted that the high number of false negatives due to several factors such as methodological disadvantages, disease stages, and methods for specimen collection might delay diagnosis and disease control. Recent data have suggested that the accuracy of nucleic acid testing is about 30–50%, approximately [4, 7, 8]. Using CT imaging feature extraction, we were able to achieve more than 89.5% accuracy, significantly outplaying nucleic acid testing. More interestingly, in testing CT images from COVID-19 patients when initial pathogenic testing was negative, our model achieved an accuracy of 85.2% for correctly predicting COVID-19. According to a study authored by Xia et al, 75% of patients with negative RT-PCR results demonstrated positive CT findings [21]. The study recommended chest CT as a primary tool for current COVID-19 detection.

Deep learning methods have been used to solve data-rich biology and medicine problems. A large amount of labelled data are required for training [22]. Although we are satisfied with the initial results, we believe that higher accuracy can be achieved by including more CT images in the training. Therefore, further optimization and testing of this system are warranted. To achieve this, we generated a webpage that licensed healthcare personnel can access, to upload CT images for testing and validation. The webpage can be accessed using https://ai.nscc-tj.cn/thai/deploy/public/pneumonia_ct.

Since the COVID-19 outbreak, several CNN models based on conventional feature extraction have been studied for COVID-19 screening from CT images. For example, Yang and colleagues used CNN and analyzed 152 manually annotated CT images and obtained an accuracy of 0.89 in COVID-19 diagnosis; however, data from this study were acquired from embedded figures on PDF files of preprints, and the validation sets were relatively small [23]. Khater and colleagues reported an accuracy of 96% using a composite hybrid feature extraction and a stack hybrid classification system in distinguishing between severe acute respiratory syndrome (SARS) and COVID-19 from 51 CT images, and this method showed a better performance than using the DCNN algorithm alone [24]. Nuriel also constructed a DCNN model based on MobileNetV2 to evaluate the ability of deep learning to detect COVID-19 from chest CT images and achieved an accuracy of 0.84 [25]. Moreover, Halgurd proposed a novel AI-enabled framework to diagnose COVID-19 based on smartphone embedded sensors, which can be used by doctors conveniently [26]. We compared the four published models and discussed the differences in each model. Evidently, the accuracies obtained varied, but the advantage of our model is that it distinguishes COVID-19 from other typical viral pneumonia. For example, despite the high accuracy of the model from Khater, it could only distinguish SARS and COVID-19. The two models from Yang et al and Nuriel et al obtained similar accuracies; however, they compared COVID-19 and other lung diseases. Accurately distinguishing between COVID-19 and other typical viral pneumonia, both of which have similar radiologic characteristics, is critical when COVID-19 and seasonal viral pneumonias co-exist. Moreover, our model showed continuous improvement as well as optimization.

However, our study has some limitations. Although DL was used to represent and learn predictable relationships in many diverse forms of data, and it is promising for applications in precision medicine, many factors such as low signal-to-noise ratio and complex data integration have challenged its efficacy [27]. CT images represent a difficult classification task due to the relatively large number of variable objects, specifically the imaged areas outside the lungs that are irrelevant to the diagnosis of pneumonia [12]. In addition, the training dataset is relatively small. The performance of this system is expected to increase when the training volume is increased. Notably, the features of the CT images we analyzed were from patients with severe lung lesions at later stages of disease development. Although we enrolled 15 patients with COVID for assessing the value of the algorithm for early diagnosis, a larger number of databases to associate this with the disease progress and all pathologic stages of COVID-19 are necessary to optimize the diagnostic system.

In the future, we intend to link the hierarchical features of CT images to features of other factors such as genetic, epidemiological, and clinical information for multi-modelling analysis to facilitate enhanced diagnosis. The artificial intelligence developed in our study can significantly contribute to COVID-19 disease control by reducing the number of PUIs to aid timely quarantine and treatment.

## Abbreviations

AI: Artificial intelligence
AUC: Area under the curve
CI: Confidence interval
CNN: Convolutional neural network
COVID-19: Corona virus disease
CT: Computed tomography
CV: Computer vision
DCNN: Deep convolutional neural network
DL: Deep learning
FP: False positive
GGO: Ground-glass opacity
IRB: Institutional review board
M-inception: Modified inception
NPV: Negative predictive value
PPV: Positive predictive value
PUI: Person under investigation
RGB: Red green blue
ROI: Region of interest
RT-PCR: Reverse transcription-polymerase chain reaction
SARS: Severe acute respiratory syndrome
SARS-COV-2: Severe acute respiratory syndrome coronavirus 2
TP: True positive
